# Supplementary material for: Seminoma and Embryonal Carcinoma Footprints Identified by Analysis of Integrated Genome-Wide Epigenetic and Expression Profiles of Germ Cell Cancer Cell Lines
Source: PLoS One. 2014 Jun 2;9(6):e98330. doi: 10.1371/journal.pone.0098330 (PMC4041891; doi:10.1371/journal.pone.0098330)
Supplement: File S1 — ZIP file containing DMRforPairs output for significant regions. Please start from the html files. (ZIP) [file pone.0098330.s008.zip › figures/19403.pdf]

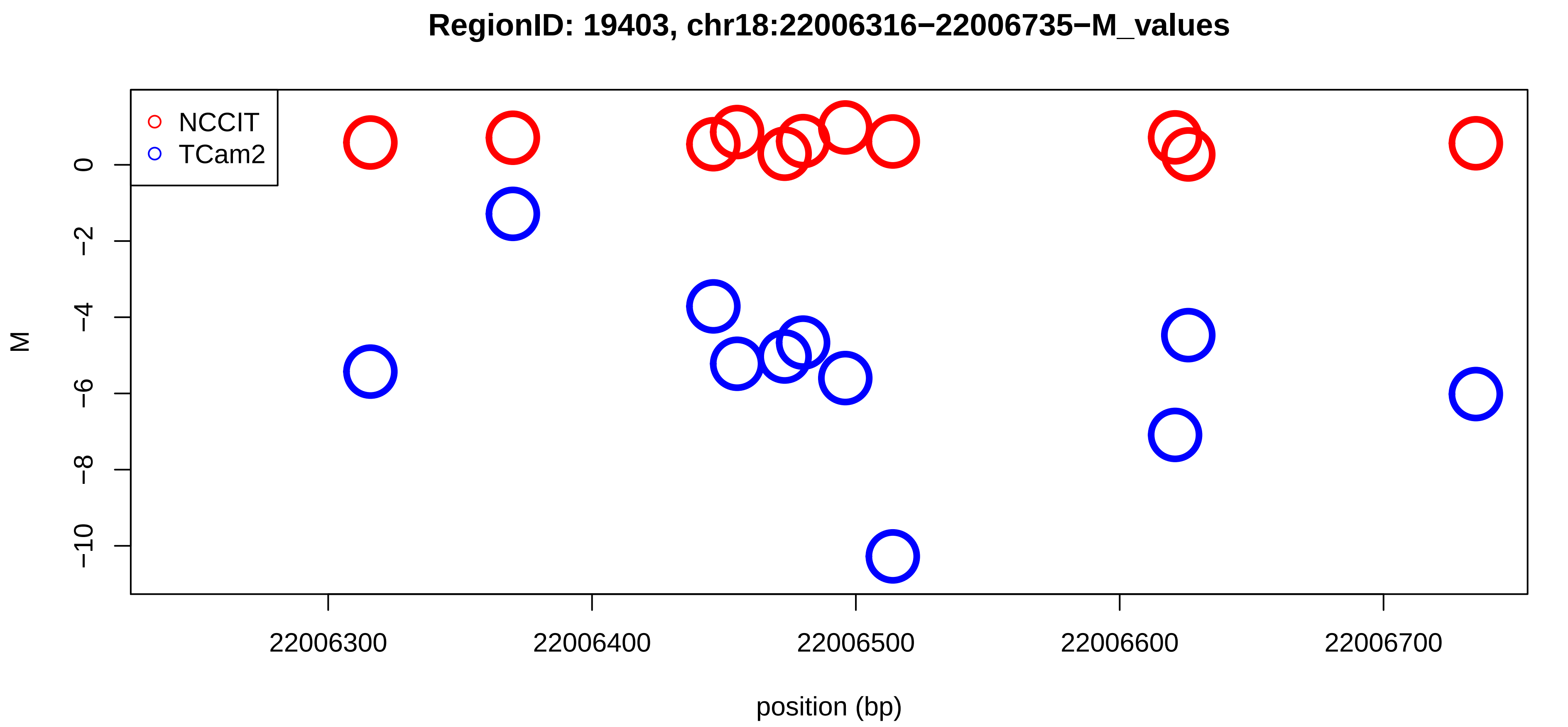
RegionID: 19403, chr18:22006316−22006735−M_values
NCCIT
TCam2
M
0
−2
−4
−6
−8
−10
22006300
22006400
22006500
22006600
22006700
position (bp)

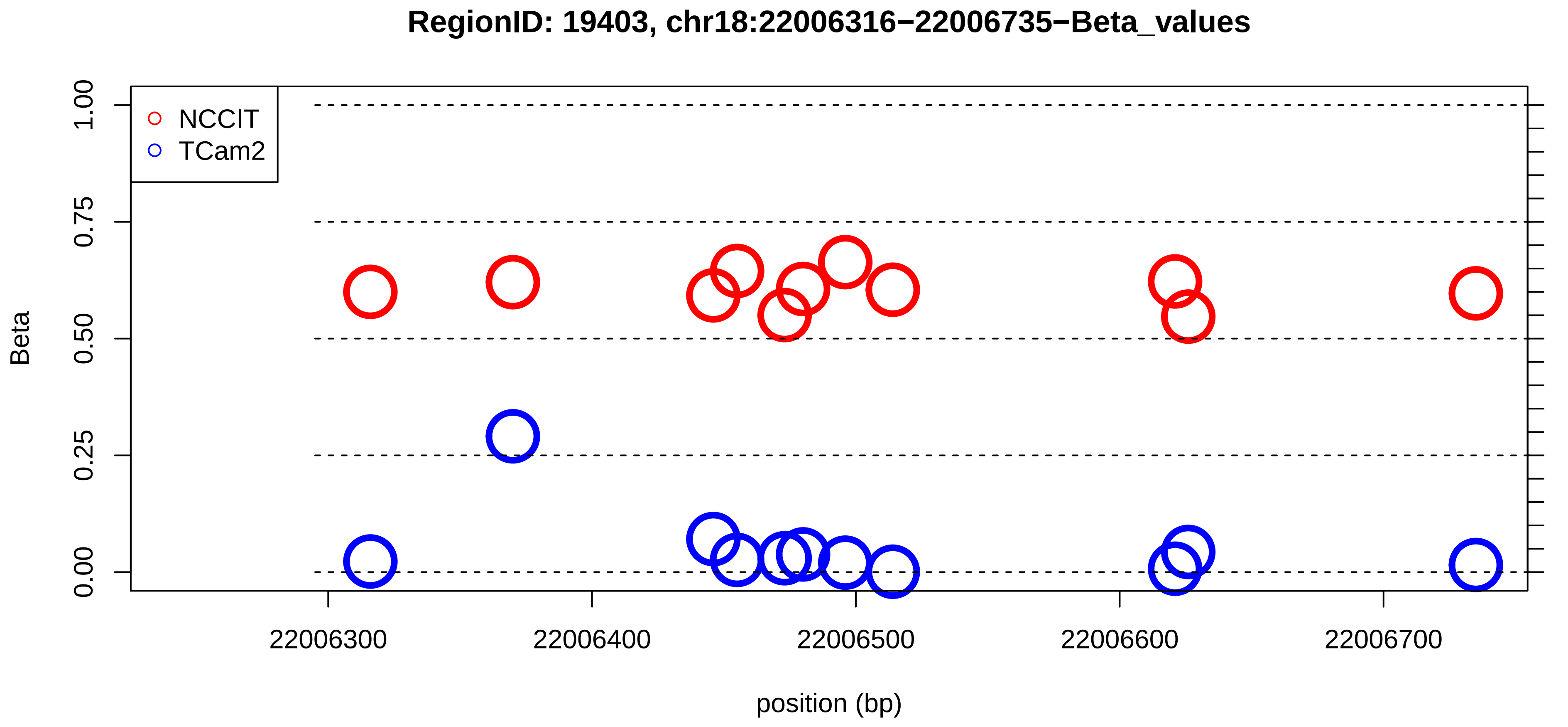
RegionID: 19403, chr18:22006316−22006735−Beta_values
NCCIT
TCam2
Beta
1.00
0.75
0.50
0.25
0.00
22006300
22006400
22006500
22006600
22006700
position (bp)

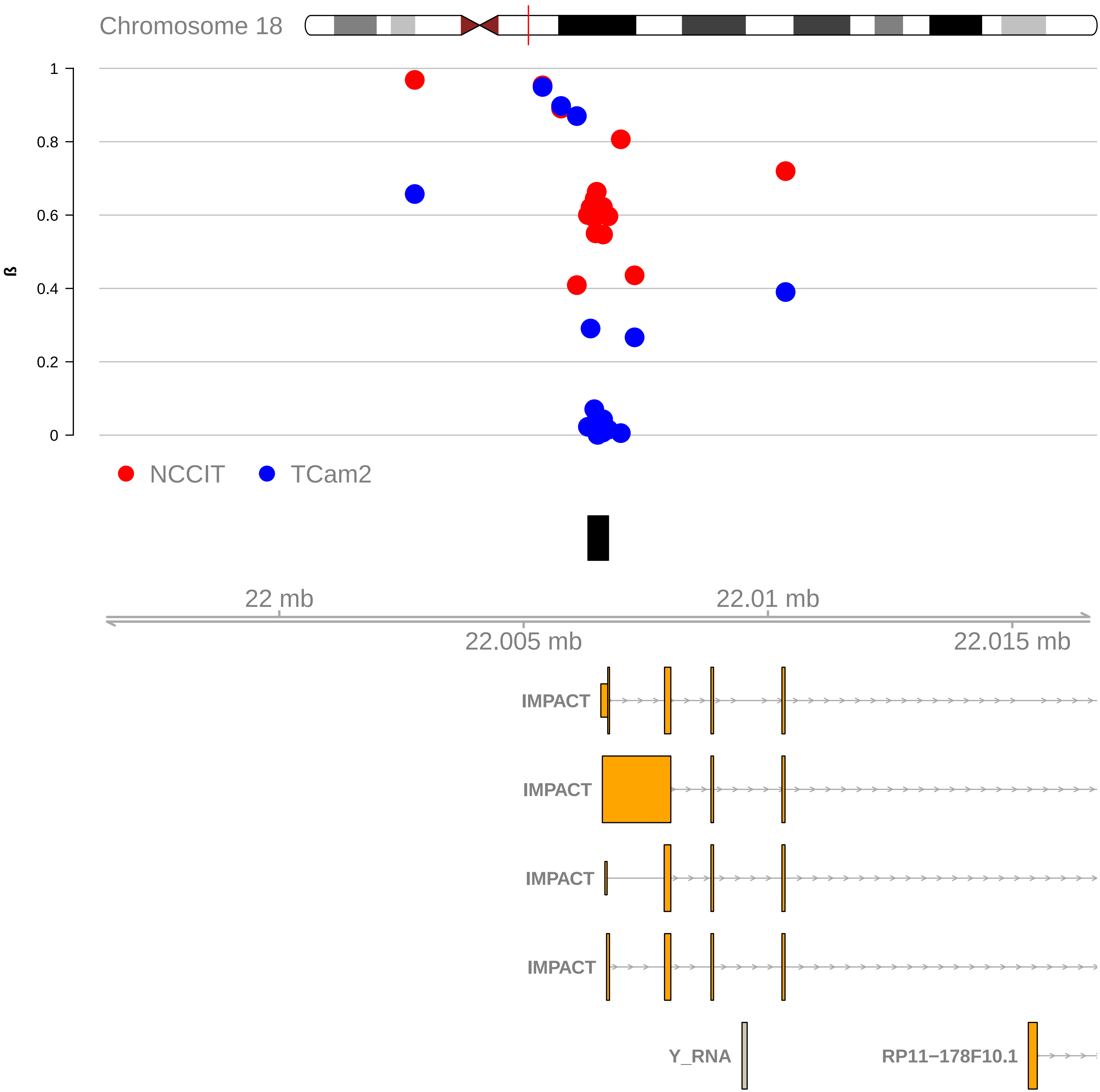

Chromosome 18
1
0.8
0.6
0.4
0.2
0
ß
NCCIT
TCam2
22 mb
22.005 mb
22.01 mb
22.015 mb
IMPACT
IMPACT
IMPACT
IMPACT
Y_RNA
RP11−178F10.1
